# Redistribution of elements of metals in plant tissues under treatment by non-ionic colloidal solution of biogenic metal nanoparticles

**DOI:** 10.1186/1556-276X-9-354

**Published:** 2014-07-15

**Authors:** Nataliya Taran, Ludmila Batsmanova, Yevheniya Konotop, Alexander Okanenko

**Affiliations:** 1 Educational and Scientific Center, Institute of Biology, Taras Shevchenko National University of Kyiv, Kyiv 03022, Ukraine

**Keywords:** Metal nanoparticles, Wheat, Seed treatment, Foliar treatment, Metal accumulation

## Abstract

The content of metal elements in plant tissues of 10-day wheat seedlings after seed pre-treatment and foliar treatment with non-ionic colloidal solution of metal nanoparticles (Fe, Mn, Cu, Zn) was determined by an atomic absorption spectrometer. It was shown that metal nanoparticles due to their physical properties (nanoscale and uncharged state) were capable of penetrating rapidly into plant cells and optimizing plant metabolic processes at the early stages of growth and development.

## Background

Nanomaterials and nanotechnology are used in all sectors of agriculture nowadays. The use of nanotechnology in agriculture (for growing grains, vegetables, and plants and for raising animals) and food production (the processing and packing) will lead to the creation of an entirely new class of food - ‘nano,’ which will eventually displace the market of genetically modified products [1]. The application of such nanoproducts as micronutrients in agriculture results in the fact that resistance to adverse climatic conditions and yields of main agrarian and technical cultures increase twofold more on the average [2]. Bioactive iron nanoparticles can increase yields of some crops up to 40% [3]. A positive impact of nanoscale magnesium upon photosynthesis productivity is also expected [4]. Achievements of nanotechnology are currently applied after harvesting sunflower, tobacco, and potatoes and in storing apples [5]. Nanopreparations possess several advantages over traditional solutions: they are not stratified by heat and light and ready-made working solution can be stored for years remaining active. But the most important point is that nanoscale preparations ensure complete wetting of the plant surface. They are completely absorbed by plants and not washed away by rain. Their effect can be observed within 2 h after application, while the action of ordinary foliarly used substances is marked within 6 to 8 h. Although nanoemulsion is expensive, it gives a much greater effect in the end. For example, winter wheat treatment with ‘Title Duo, KRR’ can provide profitability enlarged up to 400% and an additional yield of up to 17 t per hectare [4].

A promising peculiarity of nanopreparation applications is their use in very low concentrations in order to obtain environmentally friendly products. Nanoparticles penetrate easily into animal and human cells because of their size calculated in nanometers, but a bit harder into plant cells due to the cellulose cell wall. Some years ago, scientists wondered whether nanoparticles can penetrate into seeds that have a thicker shell. There are reports in the literature concerning the ability of multiwalled carbon nanotubes to penetrate through membrane into tomato seeds [6]. There is a glaring lack of knowledge about features of penetration and translocation of metal nanoparticles into plant tissues, and the data collected are often contradictory [7].

Therefore the aim of our study was to determine the content of metal elements in plant tissues after seed pre-treatment and foliar spraying of seedlings of winter wheat with non-ionic colloidal solution of metal nanoparticles.

## Methods

Winter wheat Kyivska 8 cultivar was grown in sand culture watered with tap water. Two types of experiments were performed. During the first experiment, the seedlings were grown from seeds pre-treated with individual metal nanoparticle colloidal solutions (Fe, Mn, Cu, Zn). The seeds were soaked for 24 h in aqueous solution at the concentration of 120 mg/l. Plants were grown in sand culture at 25°C and watered with tap water (photoperiod 16 h and illumination by luminescent lamps 4,000 lx). Metal content was determined in leaves and roots of 10-day seedlings.

During the second experiment, the seedlings were grown from seeds that had been soaked for 24 h in an aqueous mixture of the same metal nanoparticles and 10-day seedlings grown from non-treated seeds were sprayed with the same mixture. Samples were taken in 24 h after spraying. Colloidal solutions of metal nanoparticles were developed by the Technology of Structural Materials and Material Science Department of the National University of Life and Environmental Sciences of Ukraine and obtained as a result of dispersing iron, copper, manganese, and zinc granules by pulses of electric current with an amplitude of 100 to 2,000 A in water [2]. One control option was soaking seeds in distilled water for 24 h, and the other option was spraying the aboveground parts of seedlings with water. Metal content in the roots and aboveground parts (leaves) in 10-day wheat seedlings was determined by atomic absorption spectrometer equipped with an acetylene torch and a set of spectral lamps according to generally accepted technique [8]. Statistical analysis of the data was performed by analysis of variance (ANOVA). The reliability of the differences between the variants was assessed by Student's test at a significance level of *P* < 0.05.

## Results and discussion

**Figure 1:**
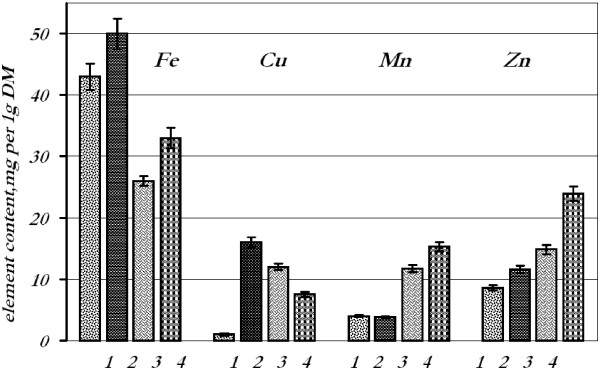
**Content of metal elements in wheat seedling tissues after treatment with individual metal nanoparticles.** 1 - roots, control; 2 - roots, experiment; 3 leaves, control; 4 - leaves, experiment.

Results obtained for seeds treated with the solution of individual metal nanoparticles showed that various elements distributed differently in the tissues of roots and leaves of seedlings (Figure 1). Thus, treatment of seeds by iron nanoparticles caused its content increase in roots and leaves of seedlings by 16 and 26%, respectively. Similarly, zinc accumulation in roots increased by 34% and in leaves by 61%. Copper content went up after treatment by copper nanoparticles in roots (by 94%); however, in leaves, it decreased (by 38%). The content of manganese increased (by 30%) in leaves of treated plants and remained at control level in the roots.

**Figure 2:**
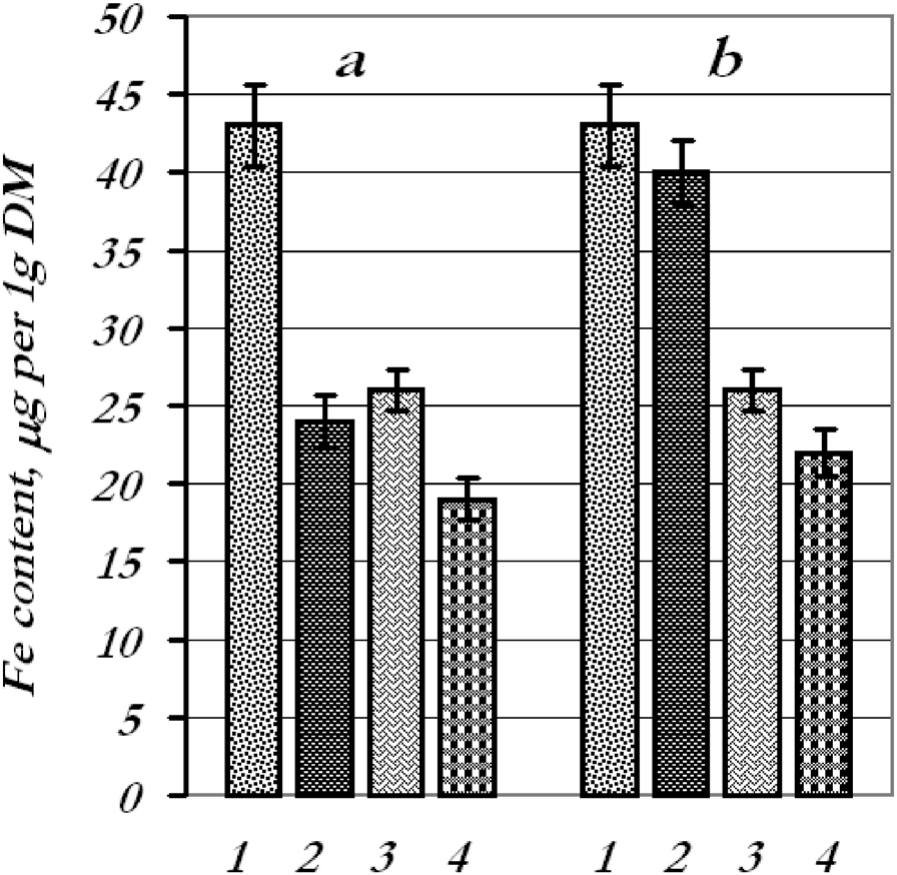
**Content of iron in wheat seedling tissues.** Iron content in tissues after treatment of seeds **(a)** and leaves **(b)** with the mixture of metal nanoparticles: 1 - roots, control; 2 - roots, experiment; 3 - leaves, control; 4 - leaves, experiment.

**Figure 3:**
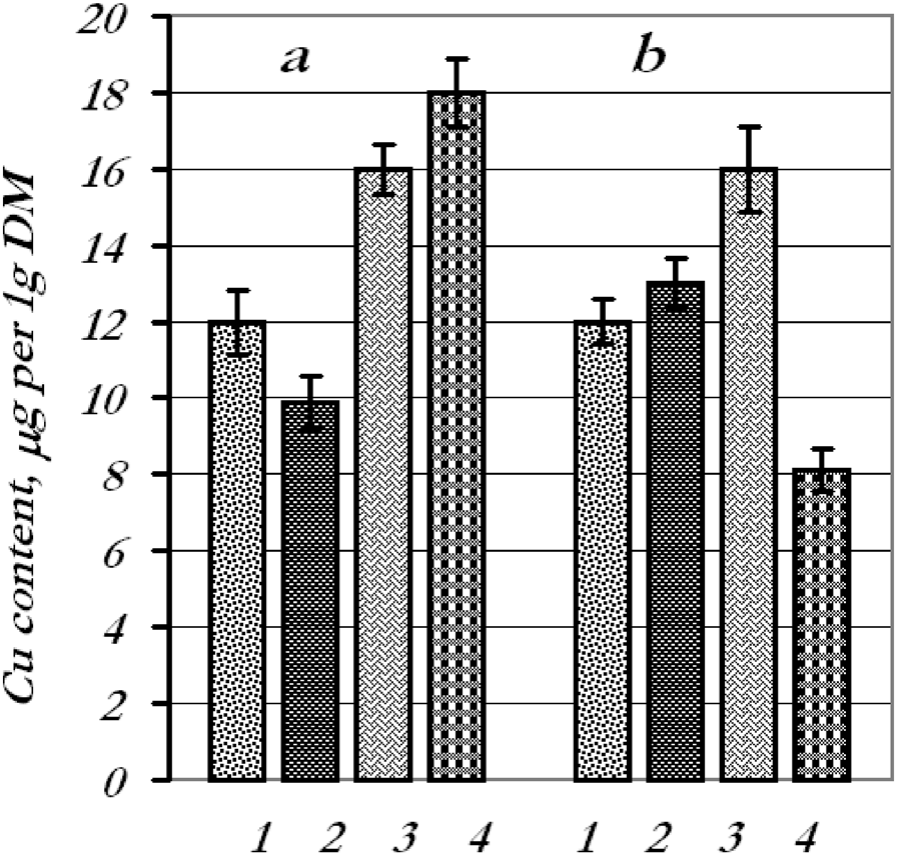
**Content of copper in wheat seedling tissues.** Copper content in tissues after treatment of seeds **(a)** and leaves **(b)** with the mixture of metal nanoparticles: 1 - roots, control; 2 - roots, experiment; 3 - leaves, control; 4 - leaves, experiment.

**Figure 4:**
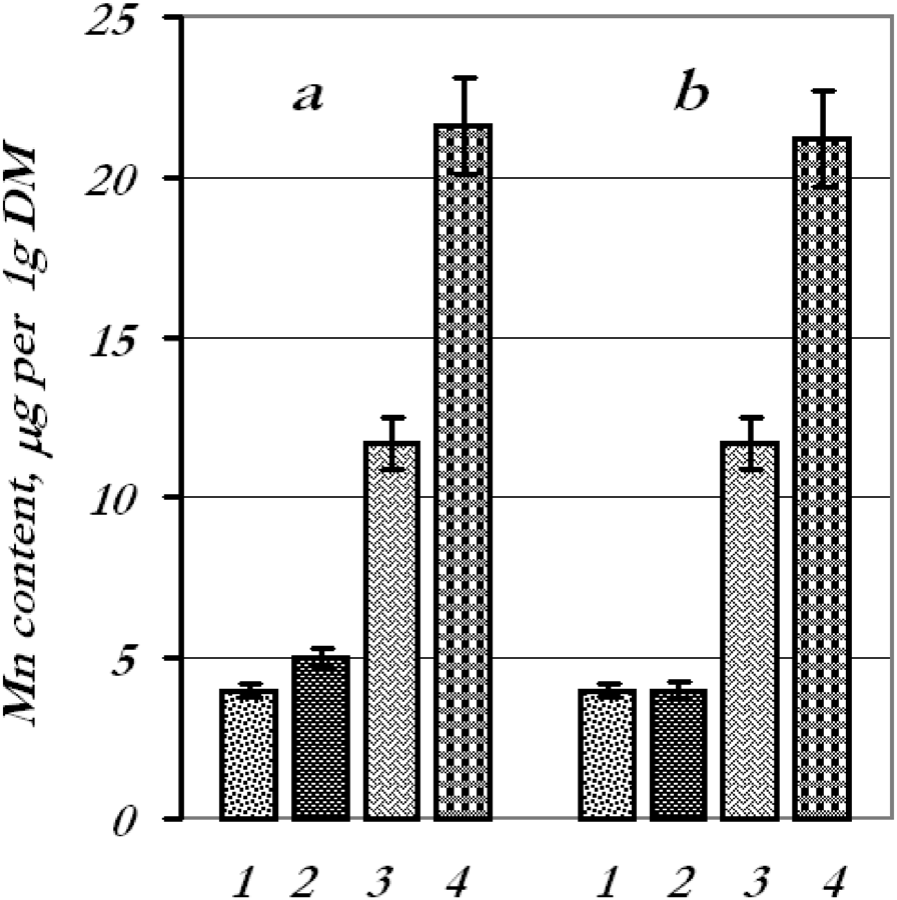
**Content of manganese in wheat seedling tissues.** Manganese content in tissues after treatment of seeds **(a)** and leaves **(b)** with the mixture of metal nanoparticles: 1 - roots, control; 2 - roots, experiment; 3 - leaves, control; 4 - leaves, experiment.

**Figure 5:**
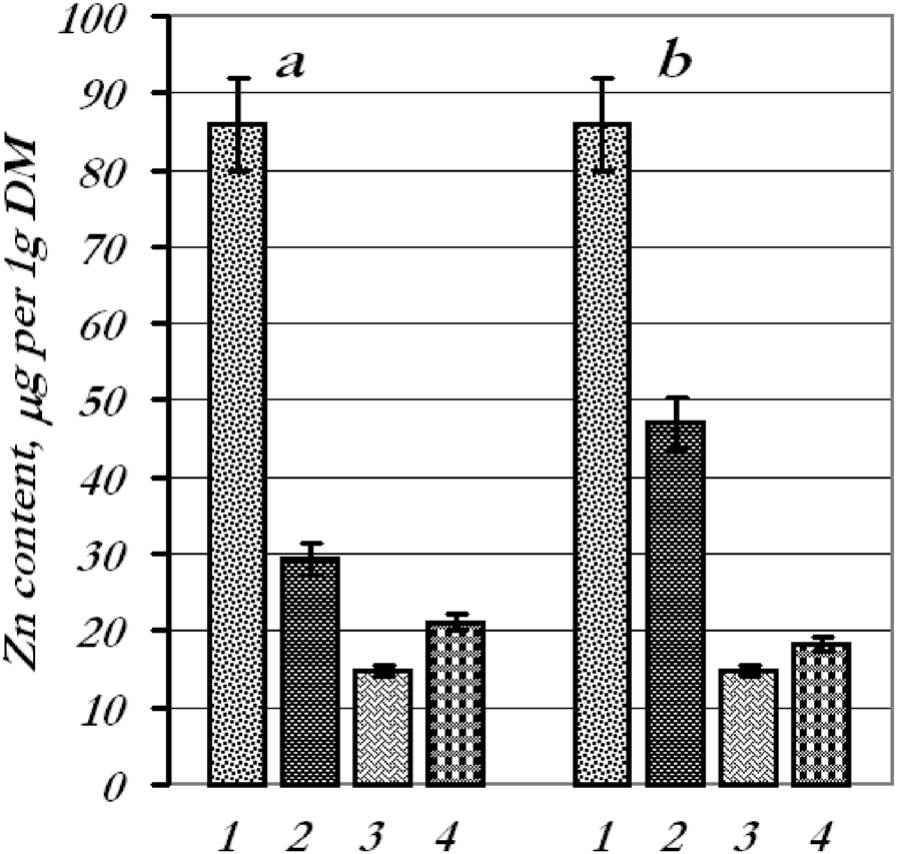
**Content of zinc in wheat seedling tissues.** Zinc content in tissues after treatment of seeds **(a)** and leaves **(b)** with the mixture of metal nanoparticles: 1 - roots, control; 2 - roots, experiment; 3 - leaves, control; 4 - leaves.

Thus, the results indicate the ability of metal nanoparticles to penetrate through the seed coat. The distribution of elements in plant tissues is determined by their ability to penetrate and peculiarities of transporting in the plant. Concerning the mechanism of processes, we could assume that nanoparticles with diameter less than the pore diameter of the cell wall could easily pass through and reach the plasma membrane [9]. After entering the cells, the nanoparticles transport from one cell to another through plasmadesmata. Major cell wall components are carbohydrates which are linked to form a rigid complex network and proteins [10]. The functional groups, such as carboxylate, phosphate, hydroxyl, amine, sulfhydryl, and imidazole, contained in these biomolecules offer a range of distinct active sites [11].We investigated both the mixtures of nanoparticle solutions and the way of their application (pre-sowing treatment and spraying of aboveground plant parts) impact upon metal contents in plant roots and leaves (aboveground parts) (Figures 2,3,4 and 5).

After seed treatment with a mixture of metal nanoparticles with subsequent determination of the content of certain metals in the leaves and roots, we found that the iron content decreased in the roots (44%) and in the leaves (27%), copper content decreased in the roots (17.5%) while in the leaves increased by 12.5%, manganese content went up in the roots (25%) and leaves (84%), and zinc content decreased drastically in the roots (66%) and increased in the leaves (42%).

Contents of iron, copper, and manganese in the roots remained at control options after foliar spraying with the mixture of metal nanoparticles; however, iron and copper contents in the leaves decreased by 15% and 49%, respectively, and manganese increased by 81%. The quantity of zinc in the roots decreased by 45%, whereas in the leaves, it went up by 23%.

Thus, we faced the phenomenon of nanoparticle antagonism for iron and zinc (in the roots) when they were applied in mixture. It could be perhaps explained by aggregation of nanoparticles or toxic effects during the combined application. Manganese accumulation might be connected presumably with a photosynthetic apparatus.

Foliarly applied substances, aqueous solutions of trace element salts, which are used for foliar feeding, are becoming more common nowadays. The permeability of these micronutrients through the leaf cuticle is limited by electrochemical potential and incomplete salt solubility. Using uncharged elements with smaller size including metal nanoparticles will improve the efficiency of micronutrients. The fact that nanoparticles passed through the epidermal cell wall opens the possible application of these nanotechnology tools for agronomical purposes. Nanoparticles applied on leaf surfaces could also pass through the stomatal openings or through the bases of trichomes and then translocate to various tissues [12,13]. Concerning their internalization in metabolism studies of dispersed phases, showed that nanoparticle solutions also contain the oxide nanoparticles, the H_2_O molecules, and the hydroxyl group-OH which surround metal particles. Nanoparticles due to their small size can contact with nucleic acids (causing, particularly, the formation of adducts of DNA) and proteins embedded in the membrane and can penetrate the cellular organelles and thus change function of biostructures. Further internalization occurs during endocytosis with the help of a cavity-like structure formed around the nanoparticles by plasma membrane and then translocated to various tissues [14]. They may also cross the membrane using embedded transport carrier proteins or through ion channels. In the cytoplasm, the nanoparticles may bind with different cytoplasmic organelles and interfere with the metabolic processes at that site [15]. By ion transportation or secretion of proteins and other biological molecules, a cell can transform a binding nanoparticle surface into something very different from that initially placed into the system. Thus, the nano-biointerface is dynamically changing until a thermodynamically favorable energy state is reached [16].

## Conclusions

Thus, the results obtained indicate the ability of metal nanoparticles to penetrate through the seed coat. The effect of application depends upon nanoparticle composition in the solution and the way of treatment. The distribution of elements in plant tissues is determined by their ability to penetrate and peculiarities of transporting in the plant. Penetration of metal nanoparticles occurs through the epidermis and stomata of aerial plant parts under treatment with nanofertilizer. Nanoparticles of metals are quickly transported through the plant and included in the metabolic processes. Fluctuation of content of individual metal elements in plant tissues may be associated with metabolic regulation of homeostasis at the cell level, namely, with the ability of nanoparticles to optimize the metabolic processes; thus, the content of elements increases in tissues where activity of metals is necessary because the elements studied are part of the organic molecules, such as enzymes. Besides, possible nanoparticle antagonism in the case of mixture application should be taken into account. The results indicate that the metal elements are not accumulated in plant tissues, which is ecologically essential for crop production.

## Competing interests

The authors declare that they have no competing interests.

## Authors’ contributions

NT conceived the study and participated in its design and coordination. LB carried out the determination of metal content in the leaves and roots of plants. YK participated in the design of the study and conducted two types of experiments in sand culture and performed the statistical analysis. AO drafted the manuscript. All authors read and approved the final manuscript.
